# Job Satisfaction of Health Practitioners Providing Outreach Health Services during COVID-19 in Rural New South Wales (NSW) and the Australian Capital Territory (ACT), Australia

**DOI:** 10.3390/healthcare11010003

**Published:** 2022-12-20

**Authors:** Md Irteja Islam, Sharif Bagnulo, Yiwen Wang, Robyn Ramsden, Trent Wrightson, Amanda Masset, Richard Colbran, Mike Edwards, Alexandra Martiniuk

**Affiliations:** 1 Sydney School of Public Health, Faculty of Medicine and Health, The University of Sydney, Camperdown, Sydney, NSW 2006, Australia; 2 Centre for Health Research and Faculty of Health, Engineering and Sciences, The University of Southern Queensland, West Street, Darling Heights, Toowoomba, QLD 4350, Australia; 3 NSW Rural Doctors Network, Suite 1, 53 Cleary Street, Hamilton, Sydney, NSW 2303, Australia; 4 Australasian College of Health Service Management, 11/41-43 Higginbotham Rd., Gladesville, Sydney, NSW 2111, Australia; 5 School of Health and Social Development, Deakin University, 1 Gheringhap Street, Geelong, Melbourne, VIC 3220, Australia; 6 Office of the Chief Scientist, The George Institute for Global Health, Level 5/1 King Street, Newtown, Sydney, NSW 2042, Australia; 7 Dalla Lana School of Public Health, The University of Toronto, 155 College Street Room 500, Toronto, ON M5T 3M7, Canada

**Keywords:** outreach health services, health practitioners, health workforce, job satisfaction, rural, COVID-19, pandemic, health professionals, telehealth, communication, capability, referral

## Abstract

**Background:** Outreach health practitioners play a key role in enhancing access to healthcare for remote, rural, regional, and Aboriginal and Torres Strait Islander communities in Australia. Outreach health practitioners are those providing ongoing and integrated health services in communities that would otherwise have limited access. In the context of the COVID-19 pandemic, it is important to understand the job satisfaction of health workers as it correlates with long-term retention of the workforce, as well as effectiveness in the role and clinical outcomes for patients. **Method:** The study analysed data from 258 outreach health practitioners who responded to two cross-sectional surveys conducted by the NSW Rural Doctors Network during the COVID-19 pandemic in 2020/21 and 2021/22 in NSW and the ACT, Australia. Both bivariate and multivariate analyses were employed to assess the associations between the outcome variable (outreach health practitioners’ job satisfaction) and independent variables (sociodemographic factors, motivation, self-confidence, communication, capability). **Results:** Overall, the study showed that 92.2% of health practitioners were satisfied in their role providing outreach health services during the COVID-19 pandemic. In the multivariable model, factors significantly associated with higher satisfaction included good communication with other local health practitioners, using telehealth along with in-person care, and having high self-rated capability compared to those health practitioners who said they had lower job satisfaction. **Conclusions:** Outreach health practitioners’ job satisfaction is important because poor satisfaction may lead to suboptimal healthcare delivery, poor clinical outcomes, and poor retention of staff in rural settings. These findings should be taken into consideration when developing future strategies to improve job satisfaction among rural outreach health practitioners and to enhance attraction, recruitment and retention and may be applicable to the broader health workforce.

## 1. Introduction

Health practitioner shortages, burnout and uneven distribution of health workers have always been important factors in healthcare delivery in both high-income, and low- and middle-income countries [1,2,3,4,5]. Rural populations are more affected by health workforce shortages affecting the ability to provide equitable access to health services [6,7]. The COVID-19 pandemic has exerted further pressure on all health systems, yet it has been argued that pre-existing unmet healthcare needs in rural and remote settings are exacerbating pandemic risks in these settings [8]. 

Rural Australians live with more chronic diseases, have more injuries, experience poorer mental health, and cannot easily access healthcare (including primary and allied health, specialist, and hospital care) compared to those living in urban areas [9,10,11]. Geographical distance alone does not cause weaker health systems. Remote and rural settings often experience reduced economic opportunities, socio-economic disadvantage, and comparatively less political power [10,12]. The vast geography typical of rural settings often increases the difficulty of accessing health services through the time and disruption to life associated with seeking or delivering healthcare. As well, limited access, costly, and lengthy transport are some of the real barriers to delivering and seeking healthcare in rural settings. Out-of-pocket fees for a health professional which might be charged in an urban setting are less likely to be affordable to rural people, on average. In Australia, nearly 28% of the Indigenous population lives in rural areas, where they have a higher burden of disease and death than urban populations [13,14,15]. The poorer health and health outcomes for Indigenous populations reflect the impacts of colonisation, forced dispossession, stolen generations, intergenerational trauma, discrimination, and racism impacting upon social determinants of health including education, employment, housing, and access to healthcare. In Australia, there is a growing understanding that enhancing cultural safety for Aboriginal and Torres Strait Islander patients may enhance both patient access and the standard of care. This refers to a health system that addresses racism and inequalities while respecting Indigenous cultural values, strengths, and uniqueness [16]. Therefore, a large proportion of health outreach services in New South Wales (NSW) and the Australian Capital Territory (ACT) in Australia are specifically for Aboriginal communities to enhance access to culturally safe health services. 

There are many contributors to efforts to improve rural and remote health, including federal and state governments, as well as non-governmental organisations (NGOs). For instance, the Australian federal government has been implementing a wide range of policy measures for more than two decades [14,15,17]. Some of these measures include investing in training and development for healthcare professionals to increase the rural health workforce. In addition to supporting university-based research to build a better understanding of the challenges of rural health and improve the evidence base for addressing these challenges [10,14,15,18,19]. Increasing the numbers of healthcare workers is typically not sufficient, but instead there is a need to focus on improving recruitment, distribution, retention, and productivity of healthcare workers to help improve health system performance [7]. There are yet further factors which influence each of these. It is known that the job satisfaction of health workers influences health service quality, as well as staff retention and productivity [20,21]. The COVID-19 pandemic has also highlighted the importance of ensuring the safety of healthcare workers while at work, in terms of workplace culture, safe workload and physical safety, such as having sufficient personal protective equipment [22]. There is a dire need to strengthen the overall system of healthcare to relieve the burden on areas of care which are particularly overloaded—such as emergency departments, paramedic services, obstetrics and maternal services and primary care [23].

### 1.1. Outreach Health Services

Outreach services have been recognised by the WHO [7] as a viable health service delivery model to maintain health services for rural populations. Within the outreach model, health practitioners travel away from their usual workplace on a regular basis to provide services to underserved areas for a few days but without permanently relocating to rural areas [24]. Outreach services can also increase attraction and retention of other, more permanent, health workers in underserved areas through reducing professional isolation, reducing workload, increasing supervision and feelings of being valued [25]. The Australian National Outreach Policy, which originated in 2000, subsidises doctors, allied health practitioners, nurses and Aboriginal health practitioners who deliver outreach health services in rural and Aboriginal health settings that are different from their usual place of practice. The ‘Medicine in Australia: Balancing Employment and Life (MABEL)’ cross-sectional survey study in Australia in 2014 found that 19% of medical specialists were practicing in rural outreach services; noting this study had a 22% response rate [24,26]. A study by Gruen et al. [11] showed outreach services reduced the average cost of consultations and reduced hospital admissions.

Outreach services can be implemented in various ways. This can impact on healthcare service acceptability, continuity, sustainability and health worker job satisfaction and retention [27,28,29]. For instance, in studies of the stability of some types of rural outreach services in Australia, it was found that only half of the health professionals provided services to the same town for at least three years [26,27]. Some outreach services are provided by different healthcare professionals with resultant loss in continuity of care for the local community, as well as placing further pressure on permanent staff in terms of training and supporting these short-term health services supports [30,31]. Some outreach models are not coordinated with local services, and this results in a disinvestment in local full-time services. 

In comparison, outreach services which are implemented long-term, with a focus on consistency of healthcare professionals, integrated with the local system and hiring local staff where possible, coordinating care, including referred services provided by medical specialists or allied health practitioners, may increase quality of care as well as job satisfaction for providers and may lead to greater retention in the role [11]. The Rural Doctor Network outreach services are implemented in a unique and deliberately integrated way and may not have the same kind of turnover as other models of outreach service delivery.

### 1.2. NSW Rural Doctors Network’s Outreach Program Model

The Rural Doctors Network (RDN) administers the Australian Government’s funded outreach programs in New South Wales (NSW) and the Australian Capital Territory (ACT). Examples of RDN-administered outreach programs are the Rural Health Outreach Fund (RHOF), Medical Outreach Indigenous Chronic Disease Program (MOICDP), Visiting Optometrists Scheme (VOS), Ear and Eye Surgical Support Services (EESS), Healthy Ears, Better Hearing, Better Listening (HEBHBL) and Follow-up Ear and Hearing Health Service (FEHHS).

RDN’s Outreach Program is unique in its decentralised model that directly invests in strengthening the capacity of local partner organisations, who identify priority local health needs and deliver long-term and integrated outreach services in response. These partner organisations provide local support to outreach health practitioners, and often have regional recruitment networks. In practice, the model involves funding and policy support flowing from RDN to local subcontractor organisations that play a substantial role in identifying needs, designing, and implementing services, and ongoing quality improvement. RDN’s Outreach Program subcontractor partners include Aboriginal Community Controlled Health Services (ACCHSs), regional NGOs and Local Health Districts (LHDs) that hold local expertise, knowledge, and relationships, and contribute this to outreach service design and implementation. Importantly, these local partners or organisations work with RDN to make decisions on which providers and health practitioners are contracted or employed to deliver services to best respond to their communities’ needs, as opposed to this being managed centrally. Because these local partners deliver a range of other primary care services or health programs, they are well placed to integrate outreach services with local primary health and hospital services and teams.

### 1.3. Job Satisfaction

When the health workforce has high levels of job satisfaction the quality of healthcare and patient satisfaction is enhanced [32,33]. Conversely, job dissatisfaction has also been identified as a key factor affecting the turnover of health and human services professionals [34]. It is known that, on average, health professionals in remote and rural areas are significantly less motivated and less satisfied with their jobs than health professionals working in major cities [35]. Employee dissatisfaction leads to higher employee turnover and lower performance and quality of work [36]. According to recent estimates in Australia, since 2015, the number of full-time equivalent (FTE) certified health professionals has dropped with distance. For instance, in 2020, there were 132,000 FTE physicians working in rural and/or remote areas compared to 386,000 FTE physicians working in major cities of Australia [37,38]. In Australia, ‘rural’ areas are described as geographic areas outside of major cities, sometimes known as the countryside; while ‘remote’ locations are characterised as places that are far from society [39].

More broadly, job satisfaction has a protective effect on health, happiness, subjective well-being, and self-esteem [40]. Increasing the job satisfaction of health professionals locally and those providing outreach health services can improve the attraction, recruitment, and sustainability of the rural health workforce. Currently, it is known that increased income, positive interpersonal relationships, career development opportunities, positive performance management and collaboration, employment security, and work conditions positively impact job satisfaction [35,41]. Moreover, evidence suggests development of a motivation system for the health professionals and an improvement of performance management system with non-financial incentives are associated with increased job satisfaction [42,43]. Further, electronic health records have been identified as one of the predictors of health professionals’ job satisfaction [44]. This is also associated with remote access of patient information and improvements in quality of care [44]. 

However, the factors which impact job satisfaction for rural outreach health practitioners specifically are still unknown. Additionally, there is little knowledge regarding the impacts of the COVID-19 pandemic upon outreach health services and no knowledge regarding the impact of COVID-19 on the job satisfaction of those providing outreach health services to rural communities. 

### 1.4. Impact of COVID-19 on Outreach Health Services

Moreover, SARS-CoV-2 or COVID-19 has negatively impacted health practitioners’ professional and personal lives due to its rapid spread and contagious nature [45,46,47,48]. Concerns about infection, moral distress watching patients die alone, and working under unmanageable and new (e.g., telehealth) workloads, with delayed management of conditions leading to greater complexity of disease, and furloughed staff due to COVID-19 exposure or infection resulting in reduced staffing have contributed to work stress/burnout for health professionals [45,49,50,51]. These factors have the potential to impact health practitioners’ job satisfaction [28,45,50,51]. 

### 1.5. Study Aims

Rural outreach health practitioners are a vital part of facilitating access to better quality healthcare services for rural communities. This study aimed to assess the changes to outreach health services during COVID-19, as well as the satisfaction, and factors associated with health practitioners’ satisfaction, providing rural outreach services during the COVID-19 pandemic in rural NSW and the ACT Australia following quantitative cross-sectional study design.

## 2. Methods

### 2.1. Data Source

Data for this study were drawn from two cross-sectional surveys that were conducted in 2020/21 and 2021/22 by RDN. Each year, RDN routinely surveys outreach health practitioners as part of their evaluation and quality improvement of outreach services.

**Figure 1:**
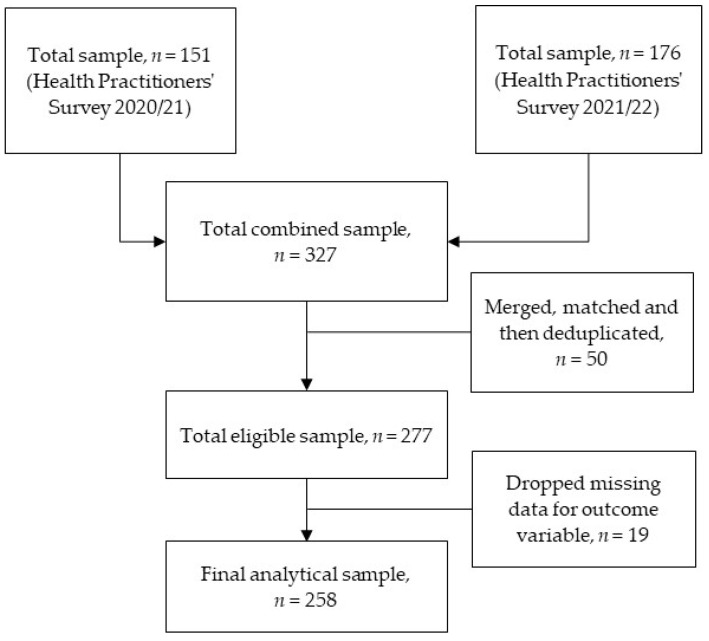
Flow diagram of sample selection.

The survey with an informed consent form was sent by email to all health practitioners who provided outreach health services via RDN during the COVID-19 pandemic in 2020 and 2021. The survey was open for ten weeks and voluntary participation was encouraged. It was sent to 845 health practitioners in the 2020/21 survey and 832 in the 2021/22 survey. In the 2020/21 outreach health practitioners survey, 151 (17.9%) responded, while 176 (21.2%) responded in the 2021/22 survey. For this study, the two survey datasets were merged and matched using a unique identification number. For any health practitioners who completed both surveys, we only included their responses to the 2020/21 survey with a thought that COVID-19 might have stronger and/or weaker impact on health practitioners’ job satisfaction and/or performance providing outreach services during the initial stage of the pandemic. Moreover, we wanted to avoid double counting the responses of one individual (i.e., weighting one individual’s beliefs more than others who only completed one survey). As well, any incomplete surveys were dropped, leading to a final number of 258 participants within this study. Figure 1 shows the flow chart for the final analytical sample selection. 

### 2.2. Measures

Health practitioners’ overall satisfaction with the outreach program was selected as the dependent variable. The following question was used to measure satisfaction: ‘How would you rate your overall satisfaction with the health outreach program?’, rated on a four-point Likert scale (very dissatisfied, dissatisfied, satisfied, very satisfied). For analytical purposes, we created a binary variable, ‘health practitioners’ satisfaction’, from the responses. Those who responded ‘satisfied’ or ‘very satisfied’ were considered as ‘satisfied’ (coded as 1), and those who replied, ‘very dissatisfied’ or ‘dissatisfied’ were labelled as ‘dissatisfied’ (coded as 0). 

Evidence suggests job satisfaction is a complicated concept relying on both internal and external factors [52,53,54]. In this study, the following variables were included as potential predictors of job satisfaction based on empirical research and availability of the variables in the dataset.

- Country of birth: Overseas or Australia
- Type of health practitioner: Aboriginal Health Worker (AHW), Nurse/Midwife, Allied health professional (e.g., Therapist, Nutritionist, Optometrist, and others) or Medical Practitioner (i.e., Specialist/GP).
- Self-confidence in being culturally responsive when treating patients: The responses were self-reported and categorised into two, somewhat confident or confident.
- Presence of motivating factors: On the basis of previous research [42,43] and context of outreach services in Australia, the survey team included the following six motivating factors—(a) To grow my practice, (b) To provide healthcare to disadvantaged people or regions, (c) To maintain a personal connection to a region, (d) To provide complex healthcare in challenging situations, (e) To provide support for rural health staff, and (f) Outreach is a requirement for employment. A five-point Likert scale was used to rank each selected motivating factor, rated from strongly disagree to strongly agree. Since high motivation has positive impact on job satisfaction and subsequently on job performance of health practitioners [42,43], we created a new variable as ‘presence of motivating factors’ from each response in this study. Categories were as follows: health practitioners with no motivating factors (coded as 0), with 1–2 motivating factors (coded as 1), with 3–4 motivating factors (coded as 2), and with 4 or more factors (coded as 3).
- Communication with other local health practitioners: A four-point Likert scale (very dissatisfied, dissatisfied, satisfied, very satisfied) was used. In this study, we created a dichotomous variable from the responses; ‘satisfied’ and ‘very satisfied’ were categorised as ‘satisfied’ (coded as 1), and while ‘very dissatisfied’ and ‘dissatisfied’ were classified as ‘dissatisfied’ (coded as 0).
- Using only paper-based referrals: Yes or No
- Using only electronic referrals: Yes or No
- Health practitioners providing telehealth: Yes or No
- Self-reported capability: The following question was used to measure capability, ‘Acknowledging that on different days we all feel (more or less) on top of our working and personal lives, in your most recent outreach visits how would you describe your overall level of capability in fulfilling your healthcare role?’, rated on a 10-point Likert scale ranged from 0-Not capable to 10-Fully capable. For the analyses, we created a new variable as ‘Self-reported capability’ from the responses, scores 0–3 was categorised as ‘Less capable’ (coded 0), scores 4–6 labelled as ‘Capable’ (coded 1) and scores 7–10 classified as ‘More capable’ (coded 2). Note that RDN used the term ‘capability’ in the context of an ‘intersection between individual capacity and ability to respond to work considering the whole of life challenges, including work, travel, family, schools, partner, education, and social options’.

### 2.3. Statistical Analyses

At first, the sample characteristics were summarised using descriptive statistics in terms of frequency (*n*) and percentages (%). Then, Pearson’s correlation coefficient matrix was created to look at the associations between the selected study variables. Bivariate analyses were then conducted to assess the independent variables and their distributions over the dependent variable (health practitioners’ satisfaction with the outreach program). Later, logistic regression models (unadjusted and adjusted) with complete cases were only used to identify the determinants of health practitioners’ satisfaction with outreach services. Adjusted multiple logistic regression included variables that had a *p*-value of <0.05 in the unadjusted model. The results of the logistic models were described as odds ratios (ORs) with 95% confidence intervals (CI) and *p*-values. 

Lastly, several diagnostic tests were performed to evaluate the assumptions of the logistic model. For instance, we used McFadden’s R^2^ [55] and goodness-of-fit test [56] for model performance, the link test [57] for model specification, and variance inflation factor (VIF) statistics [58] to assess for multicollinearity among the predictor variables in the model. All the analyses were completed using Stata/SE 14.1 (StataCorp, College Station, TX, USA). 

### 2.4. Ethical Approval

This paper uses non-identifiable, routinely collected data in accordance with the National Statement on Ethical Conduct in Human Research. We accessed, analysed, and presented the data in an anonymous format. Secondary research data of this type supports Outcome A of the University of Sydney Research Ethics Board (Supplementary File S1). Each study participant voluntarily completed the self-reported survey questionnaire with informed consent.

## 3. Results

Table 1 presents the characteristics of the healthcare worker participants. The study comprised a total of 258 outreach health practitioners, of which more than 70% were born in Australia. Approximately, half of the participants (47.7%) were Allied Health Professionals, followed by Medical Practitioners (32.5%), Nurses/Midwives (12.4%) and Aboriginal Health Workers (7.4%). More than half of the respondents did not answer the question about their self-confidence with being culturally responsive (133, 51.6%). Of those who did answer the question, 75% (94/125) said they were confident in their ability to be culturally responsive to patients. The remaining 25% (21/125) said they were somewhat confident. Most of the participants reported to have more than three motivating factors for providing outreach services (62%), and more than 60% were satisfied in the way they communicated with other local health providers. In terms of referrals, nearly 44% of the respondents used only paper-based referrals, and 22% used only electronic referrals. Almost two-thirds provided telehealth, and most (85.7%) felt they were capable in their role as an outreach health practitioner. 

**Figure 2:**
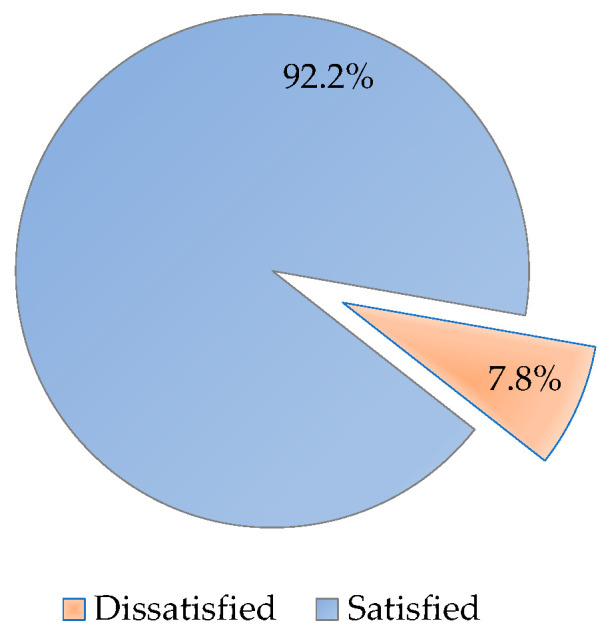
Proportion of satisfied and dissatisfied health professionals providing outreach services to rural communities in NSW and the ACT during the first 2 years of the COVID-19 pandemic.

Figure 2 illustrates the rates of overall satisfaction of the health practitioners providing outreach services. Out of a total of 258 respondents, the majority (238, 92.2%) were satisfied with the health outreach program.

Table 2 portrays the correlation coefficient matrix among the selected study variables. For example, according to the table, communication with other local healthcare providers, paper-based referrals, telehealth, and capability are positively correlated with the outcome variable; that means these variables were associated with outreach health practitioners’ level of satisfaction. 

Further, the results from the bivariate analysis between potential predictors and health practitioners’ level of satisfaction with the health outreach program are depicted in Table 3. Factors such as communication with other local health practitioners (*p* < 0.001), paper-based referral methods (*p* < 0.05), access to telehealth (*p* < 0.01) and self-reported capability (*p* < 0.01) were significantly associated with outreach health practitioners’ satisfaction during the first two years of the COVID-19 pandemic.

Table 4 presents the results of the logistic regression models (unadjusted and adjusted) that investigated the correlates of health practitioners’ overall satisfaction with the outreach health program. Statistically significant predictors (*p* < 0.05) from the unadjusted model were put into the adjusted model. In the adjusted model, outreach health practitioners who had satisfactory communication with other local health practitioners were 7.76 times (95% CI: 1.86–32.37) more likely to be satisfied with an outreach program than those who had dissatisfactory communication. Further, those who provided telehealth services were more likely to have increased satisfaction with the outreach program compared to those who did not offer telehealth (OR 4.07, 95% CI: 1.06–15.59). We also found that health practitioners who self-reported to be more capable were 10.92 times (95% CI 1.18–101.03) more likely to be satisfied with the outreach program than their counterparts.

Finally, Table 4 demonstrates the results obtained from several regression diagnostic tests. The VIF test with a mean value of 1.05 confirmed the absence of the multicollinearity in the adjusted model. McFadden’s R^2^ value of 0.263, and the insignificant goodness-of-fit statistic (*p* = 0.789), indicates the model was well-fitted. Further, the link test (*p* < 0.05) verified that the model was correctly specified.

## 4. Discussion

Job satisfaction refers to the degree to which individuals are satisfied or dissatisfied with their job in terms of their attitude toward the work they do, including the social and physical conditions at their workplace [59,60,61]. In healthcare management, job satisfaction of health workers is vital as it has been linked to improved quality of care, elevated patient compliance, and greater patient satisfaction [62,63]. It is also reported that health workers’ job satisfaction has been affected by the COVID-19 pandemic, further jeopardizing the sustainability of health services [61,64]. In this study, the overall satisfaction of health practitioners providing the RDN-administered Outreach Program services was investigated, and predictors of satisfaction were identified. The results indicate that 92.2% of respondents were satisfied with the health outreach program during the initial COVID-19 pandemic years of 2020 and 2021. Among the predictors explored in our study, communication with other local health practitioners, access to telehealth, and self-reported capability were significant factors that predicted the overall satisfaction of outreach health professionals.

### 4.1. Communication with Other Local Health Practitioners

Our study identified that outreach health practitioners who have satisfactory to good communication within the outreach team, with existing local health practitioners, as well as with practitioners being referred to, were more likely to be satisfied with the health outreach program. The link between satisfaction with communication and job satisfaction and burnout in healthcare settings has been previously identified, including in one American study completed during the first year of the COVID-19 pandemic [65,66,67,68]. Outreach health practitioners who work as temporary members of local healthcare teams may encounter time constraints, power differentials and lack of trust [69]. Communication can help build cohesive and functional teams and improve job efficiency in workplaces (Pincus, 1986). As well, good communication within a team can lead to a reduction in the number of medical errors and increase patient safety by guiding the use of technical skills within the team, thereby increasing job satisfaction [66]. Interestingly most of the existing research literature which addresses the value of communication in job satisfaction, studies physicians. For instance, Loughman et al. [67] showed that good communication in workplace decision-making can make physicians feel that they have a voice in the decision-making process. In comparison, our study included a wide range of health professionals including allied health, Aboriginal health workers and nurses/midwives as well as physicians and the finding of the importance of communication for job satisfaction remained. 

In rural practice settings, where emergencies and new challenges occur more frequently, health practitioners often have less support but more stress [11,69]. Having good relationships with colleagues not only increases employee productivity but satisfying work relationships also increase well-being and meaning in life [70]. Gardiner et al. [71] showed that by increasing opportunities for GPs and other health practitioners to discuss personal/professional issues, fewer health practitioners felt isolated or unsupported. Communication satisfaction can play a role to help outreach health practitioners achieve better performance and job satisfaction in the rural context and this has also held true in our data gathered during the initial two years of the COVID-19 pandemic. 

### 4.2. Access to Telehealth (Virtual Care)

Telehealth has been widely used during the COVID-19 pandemic to minimize infection risks [72,73]. In Australia, health practitioners used remote online delivery as well as telephone delivery of care [74,75]. For health practitioners providing outreach services, restrictions on travel, face-to-face consultations, and vaccination requirements during the first two years of the COVID-19 pandemic resulted in the delay or cancellation of health outreach services and prevented regular in-person visits [76,77]. As well, studies have shown that patients often cancelled or postponed appointments for fear of contracting COVID-19 [78,79,80]. In this study, we found telehealth services were provided at some point by almost two-thirds of healthcare professionals providing outreach care during the first two years of the COVID-19 pandemic. Moreover, the health professionals who used telehealth were more likely to be satisfied with the health outreach program. This is mainly because telehealth has allowed health practitioners to not only reduce infection risks, travel costs and time but also still help maintain continuity of service delivery during the pandemic [81,82]. It has been reported that without telehealth, health professionals are required to make in-person visits that take a lot of time to travel to see a patient, which decreases service effectiveness [82,83,84].

### 4.3. Self-Reported Capability

Health practitioners’ self-reported capability was found to be positively associated with overall satisfaction with the health outreach program in our study. Close to 86% of outreach health practitioners considered themselves as ‘more capable’ versus ‘less capable’. This might also suggest that health practitioners who perceive themselves to be more capable are more likely to participate in health outreach programs or to answer the survey. Capability is not only the mastery of the role, technology, and knowledge, but also encompasses the human emotion, body, and spirit/culture/society [85,86]. This concept stems from the capability approach developed by Amartya Sen in 1979 [87], which provides a ‘framework for assessing human well-being’ and is now also used globally in various areas including health [88]. The capability approach argues for going beyond the workplace when understanding ability to do one’s job; supplementing information about job satisfaction by looking at data on the whole life context (i.e., resources, personal and social influences) to derive an overall assessment of how well someone feels they are doing in their role(s) [89]. The focus on the social and structural elements of capability contributes to a broader understanding of workforce, including motivation, recruitment and retention, adaptation of skills in changing environments and resilience [86,90,91]. Workers can experience distress when there is a mismatch between their work challenges and their ability to develop and implement effective coping responses [92]. Supporting the capability of the health workforce can provide health practitioners with a higher level of control over problems and situations when they encounter difficulties, resulting in better job satisfaction [92]. In the literature, job satisfaction and capability are unique, yet related, concepts with satisfaction with job role being just one part of capability. In our study, we observed similar proportions of health practitioners responding that they felt satisfied to those who responded they felt capable. More research into how these terms is understood by the health workforce will be useful.

### 4.4. Limitations

This present study used cross-sectional survey data enrolling NSW and ACT outreach health practitioners working in Commonwealth funded outreach programs administered by RDN. Not all outreach health professionals completed the survey, therefore the sample may not be representative of other health outreach services. A future survey capturing data from all (or a random sample of) outreach health practitioners as well as a longitudinal assessment of factors associated with satisfaction among outreach health practitioners may be useful. A further limitation is that the data used in our study were collected across different time periods during the initial years of the COVID-19 pandemic, and we were not able to consider the potentially unique impact of differences between the initial flattening of the epidemic curve in Australia, where most rural areas experienced zero COVID-19, and the subsequent outbreak period. We were unable to confirm whether changes in the environmental context had a significant impact on changes in predictors of outreach health practitioners’ satisfaction with their role. Further, information was self-reported and may have therefore been influenced by response or social desirability bias. Moreover, the dataset was lacking some key variables such as age, sex, postcode of permanent residence, income, emotional state including any symptoms of post-traumatic stress disorder (PTSD) of the health practitioners during the COVID-19 pandemic, so we were not able to determine whether satisfaction with the role differed by these factors. A final limitation is that the questionnaire used has not been assessed for its psychometric properties, and therefore its validity and reliability are unknown.

## 5. Conclusions

This study found that nearly all the outreach health practitioners were satisfied with the RDN outreach model during the first years of the COVID-19 pandemic. It is important for outreach health practitioners to be satisfied with their jobs as it can help retain health practitioners in this role, and this is even more important during health crises such as the pandemic. It may support the quality of care provided as well as the productivity of this workforce and make rural outreach services a more attractive career option for other healthcare practitioners. We found that good communication with other local health practitioners, incorporating options for using telehealth along with in-person care and high self-reported capability, were significantly associated with higher levels of job satisfaction among rural NSW and ACT outreach health practitioners in 2020 and 2021, the initial years of the pandemic. These findings may support future strategies for health workforce support during health crises, and may be used to further enhance job satisfaction, and possibly to enhance attraction, recruitment, and retention of rural health practitioners.

## Figures and Tables

**Table 1:** Sample characteristics.

Variables	*n* (%)
**Country of birth**	
Overseas	73 (28.3)
Australia	185 (71.7)
**Type of health practitioners**	
Aboriginal Health Workers (AHWs)	19 (7.4)
Nurses/Midwives	32 (12.4)
Allied Health Professionals (AHPs)	123 (47.7)
Medical Practitioners (MP)	84 (32.5)
**Self-confidence (Cultural perspective)**	
Somewhat confident	31 (12.0)
Confident	94 (36.4)
Missing	133 (51.6)
**Presence of motivating factors**	
No factors	83 (32.2)
1–2 factors	14 (5.4)
3–4 factors	124 (48.1)
>4 factors	37 (14.3)
**Communication with local health practitioners**	
Dissatisfactory	24 (9.3)
Satisfactory	156 (60.5)
Missing	78 (30.2)
**Using paper-based referrals**	
No	146 (56.6)
Yes	112 (43.4)
**Using electronic referrals**	
No	201 (77.9)
Yes	57 (22.1)
**Provide telehealth**	
No	94 (36.4)
Yes	164 (63.6)
**Self-reported capability**	
Less capable	7 (2.7)
Capable	30 (11.6)
More capable	221 (85.7)

**Table 2:** Correlation coefficients between selected variables.

	1	2	3	4	5	6	7	8	9
1 Country of birth	1.00								
2 Type of health practitioners	−0.36 *								
3 Self-confidence	−0.01	−0.11							
4 Presence of motivating factors	0.08	−0.11	0.02						
5 Communication with local health practitioners	−0.16 *	0.15 *	−0.06	−0.01					
6 Using paper-based referrals	−0.09	−0.03	0.01	0.51 *	0.23 *				
7 Using electronic referrals	0.04	−0.05	0.06	0.34 *	0.02	0.23 *			
8 Provide telehealth	−0.06	0.11	0.07	0.03	0.06	0.20 *	0.05		
9 Self-reported capability	0.07	0.02	0.23 *	0.00	−0.04	0.02	0.02	0.02	
10 Overall satisfaction	−0.05	0.05	0.06	0.04	0.26 *	0.13 *	0.01	0.17 *	0.21 *

Level of significance: * *p* < 0.05.

**Table 3:** Factors associated with outreach health practitioners’ level of satisfaction.

	Dissatisfied	Satisfied	Pearson χ^2^ (*p*-Value)
	*n* (%)	*n* (%)	
**Country of birth**			0.73 (0.391)
Overseas	4 (5.5)	69 (94.5)	
Australia	16 (8.7)	169 (91.3)	
**Type of health practitioners**			2.60 (0.457)
AHWs	3 (15.8)	16 (84.2)	
Nurses/Midwives	3 (9.4)	29 (90.6)	
AHPs	7 (5.7)	116 (94.3)	
MPs	7 (8.3)	77 (91.7)	
**Self-confidence**			0.38 (0.538)
Somewhat confident	3 (9.7)	28 (90.3)	
Confident	6 (6.4)	88 (93.6)	
**Presence of motivating factors**			0.72 (0.868)
No factors	8 (9.6)	75 (90.4)	
1–2 factors	1 (7.1)	13 (92.9)	
3–4 factors	8 (6.5)	116 (93.5)	
>4 factors	3 (8.1)	34 (91.9)	
**Communication with local health practitioners**			13.06 (<0.001)
Dissatisfactory	6 (25.0)	18 (75.0)	
Satisfactory	7 (4.5)	149 (95.5)	
**Using paper-based referrals**			4.83 (0.028)
No	16 (11.0)	130 (89.0)	
Yes	4 (3.6)	108 (96.4)	
**Using electronic referrals**			0.05 (0.814)
No	16 (8.0)	185 (92.0)	
Yes	4 (7.0)	53 (93.0)	
**Provide telehealth**			7.63 (0.006)
No	13 (13.8)	81 (86.2)	
Yes	7 (4.3)	157 (95.7)	
**Self-reported capability**			12.20 (0.002)
Less capable	2 (28.6)	5 (71.4)	
Capable	6 (20.0)	24 (80.0)	
More capable	12 (5.4)	209 (94.6)	

**Table 4:** Determinants of outreach health practitioners’ satisfaction.

	Unadjusted OR	95% CI	Adjusted OR	95% CI	VIF
**Country of birth (Ref. Overseas)**					-
Australia	0.61	0.19–1.89	-	-	
**Type of health practitioners (Ref. AHWs)**					-
Nurses/Midwives	1.81	0.32–10.04	-	-	
AHPs	3.11	0.72–13.24	-	-	
MPs	2.06	0.48–8.84	-	-	
**Self-confidence (Ref. Somewhat confident)**					-
Confident	1.57	0.36–6.69	-	-	
**Presence of motivating factors (Ref. No factors)**					-
1–2 factors	1.38	0.15–12.03	-	-	
3–4 factors	1.54	0.55–4.29	-	-	
>4 factors	1.21	0.30–4.84	-	-	
**Communication with local health practitioners (Ref. Dissatisfactory)**					1.08
Satisfactory	7.09 **	2.14–23.44	7.76 **	1.86–32.37	
**Using paper-based referrals (Ref. No)**					1.03
Yes	3.32 *	1.07–10.23	2.38	0.59–9.58	
**Using electronic referrals (Ref. No)**					-
Yes	1.14	0.37–3.57	-	-	
**Provide telehealth (Ref. No)**					1.03
Yes	3.59 **	1.38–9.37	4.07 *	1.06–15.59	
**Self-reported capability (Ref. Less capable)**					1.04
Capable	1.60	0.24–10.36	1.72	0.13–21.50	
More capable	6.96 *	1.22–39.69	10.92 *	1.18–101.03	
**Model statistics**					
McFadden’s R^2^ (%)			0.263		
Goodness-of-fit test (*p*-value)			7.11 (0.789)		
Link test (OR for that of satisfaction)			3.79 *		
Mean VIF			1.05		

Level of significance: ** *p* < 0.01, * *p* < 0.05.

## Data Availability

The data provided in this study are accessible from the Rural Doctors Network Survey team upon request. The data are not publicly available as it is routinely collected data from health practitioners providing services at the outreach clinics as part of their annual evaluation of services and for quality improvement.

## Supplementary Materials

The following supporting information can be downloaded at: https://www.mdpi.com/article/10.3390/healthcare11010003/s1, File S1: Using Existing Data Collections in Research: Guidelines for Researchers.

## References

[1] Aith F., Castilla Martínez M., Cho M., Dussault G., Harris M., Padilla M., Murphy G.T., Tomlin P., Valderas J.M. (2020). Is COVID-19 a turning point for the health workforce?. Rev. Panam. Salud Publica.

[2] Buchan J., Campbell J. (2013). Challenges posed by the global crisis in the health workforce. BMJ Br. Med. J..

[3] Khasne R., Dhakulkar B., Mahajan H. (2020). Burnout among Healthcare Workers during COVID-19 Pandemic in India: Results of a Questionnaire-based Survey. Indian J. Crit. Care Med..

[4] Leo C., Sabina S., Tumolo M., Bodini A., Ponzini G., Sabato E., Mincarone P. (2021). Burnout among Healthcare Workers in the COVID-19 Era: A Review of the Existing Literature. Front. Public Health.

[5] Dugani S., Afari H., Hirschhorn L.R., Ratcliffe H., Veillard J., Martin G., Lagomarsino G., Basu L., Bitton A. (2018). Prevalence and factors associated with burnout among frontline primary health care providers in low-and middle-income countries: A systematic review. Gates Open Res..

[6] Retention of the Health Workforce in Rural and Remote Areas: A Systematic Review. https://apps.who.int/iris/handle/10665/337300.

[7] World Health Organization (2010). Monitoring the Building Blocks of Health Systems: A Handbook of Indicators and Their Measurement Strategies.

[8] O’Sullivan B., Leader J., Couch D., Purnell J. (2020). Rural Pandemic Preparedness: The Risk, Resilience and Response Required of Primary Healthcare. Risk Manag. Healthc. Policy.

[9] Australian Institute of Health and Welfare (2020). Profile of Indigenous Australians. https://www.aihw.gov.au/reports/australias-health/profile-of-indigenous-australians.

[10] Cosgrave C., Malatzky C., Gillespie J. (2019). Social Determinants of Rural Health Workforce Retention: A Scoping Review. Int. J. Environ. Res. Public Health.

[11] Gruen R.L., Weeramanthri T.S., Bailie R.S. (2002). Outreach and improved access to specialist services for Indigenous people in remote Australia: The requirements for sustainability. J. Epidemiol. Community Health.

[12] Wakerman J., Humphreys J., Russell D., Guthridge S., Bourke L., Dunbar T., Zhao Y., Ramjan M., Murakami-Gold L., Jones M.P. (2019). Remote health workforce turnover and retention: What are the policy and practice priorities?. Hum. Resour. Health.

[13] Australian Institute of Health and Welfare (2020). Rural and Remote Health. https://www.aihw.gov.au/reports/australias-health/rural-and-remote-health.

[14] Cosgrave C. (2020). The Whole-of-Person Retention Improvement Framework: A Guide for Addressing Health Workforce Challenges in the Rural Context. Int. J. Environ. Res. Public Health.

[15] Humphreys J., Wakerman J. (2018). Learning from history: How research evidence can inform policies to improve rural and remote medical workforce distribution. Aust. J. Rural Health.

[16] Australian Institute of Health and Welfare (2022). Cultural Safety in Health Care for Indigenous Australians: Monitoring Framework. https://www.aihw.gov.au/reports/indigenous-australians/cultural-safety-health-care-framework.

[17] McGirr J., Seal A., Barnard A., Cheek C., Garne D., Greenhill J., Kondalsamy-Chennakesavan S., Luscombe G.M., May J., McLeod J. (2019). The Australian Rural Clinical School (RCS) program supports rural medical workforce: Evidence from a cross-sectional study of 12 RCSs. Rural Remote Health.

[18] Lyle D., Greenhill J. (2018). Two decades of building capacity in rural health education, training and research in Australia: University Departments of Rural Health and Rural Clinical Schools. Aust. J. Rural Health.

[19] Mason J. (2013). Review of Australian Government Health Workforce Programs. http://www.health.gov.au/internet/main/publishing.nsf/Content/review-australian-government-health-workforce-programs.

[20] Stanković A., Nikolić M., Nikic D.S., Arandjelović M. (2008). Job Satisfaction in Health Care Workers. Acta Med. Median..

[21] Bimpong K., Khan A., Slight R., Tolley C.L., Slight S.P. (2020). Relationship between labour force satisfaction, wages and retention within the UK National Health Service: A systematic review of the literature. BMJ Open.

[22] Søvold L., Naslund J., Kousoulis A., Saxena S., Qoronfleh M., Grobler C., Münter L. (2021). Prioritizing the Mental Health and Well-Being of Healthcare Workers: An Urgent Global Public Health Priority. Front. Public Health.

[23] New South Wales Parliament, Legislative Council (2022). Portfolio Committee No. 2—Health (Report No. 57). Health Outcomes and Access to Health and Hospital Services in Rural, Regional and Remote New South Wales. https://www.parliament.nsw.gov.au/lcdocs/inquiries/2615/Report%20no%2057%20-%20PC%202%20-%20Health%20outcomes%20and%20access%20to%20services.pdf.

[24] O’Sullivan B.G., Stoelwinder J.U., McGrail M.R. (2017). Specialist outreach services in regional and remote Australia: Key drivers and policy implications. Med. J. Aust..

[25] Roodenbeke E.D., World Health Organization (2011). Outreach Services as a Strategy to Increase Access to Health Workers in Remote and Rural Areas: Increasing Access to Health Workers in Rural and Remote Areas.

[26] O’Sullivan B.G., Joyce C.M., McGrail M.R. (2014). Adoption, implementation and prioritization of specialist outreach policy in Australia: A national perspective. Bull. World Health Organ..

[27] O’Sullivan B., McGrail M., Stoelwinder J. (2017). Reasons why specialist doctors undertake rural outreach services: An Australian cross-sectional study. Hum. Resour. Health.

[28] Alrawashdeh H., Al-Tammemi A., Alzawahreh M., Al-Tamimi A., Elkholy M., Al Sarireh F., Abusamak M., Elehamer N.M., Malkawi A., Al-Dolat W. (2021). Occupational burnout and job satisfaction among physicians in times of COVID-19 crisis: A convergent parallel mixed-method study. BMC Public Health.

[29] Hussain R., Maple M., Hunter S., Mapedzahama V., Reddy P. (2015). The Fly-in Fly-out and Drive-in Drive-out model of health care service provision for rural and remote Australia: Benefits and disadvantages. Rural Remote Health.

[30] Busbridge M., Smith A. (2015). Fly in/fly out health workers: A barrier to quality in health care. Rural Remote Health.

[31] Carey T.A., Sirett D., Wakerman J., Russell D., Humphreys J.S. (2018). What principles should guide visiting primary health care services in rural and remote communities? Lessons from a systematic review. Aust. J. Rural Health.

[32] Janicijevic I., Seke K., Djokovic A., Filipovic T. (2013). Healthcare workers satisfaction and patient satisfaction—where is the linkage?. Hippokratia.

[33] Huhtala M., Geurts S., Mauno S., Feldt T. (2021). Intensified job demands in healthcare and their consequences for employee well-being and patient satisfaction: A multilevel approach. J. Adv. Nurs..

[34] Aloisio L.D., Gifford W.A., McGilton K.S., Lalonde M., Estabrooks C.A., Squires J.E. (2018). Individual and organizational predictors of allied healthcare providers’ job satisfaction in residential long-term care. BMC Health Serv. Res..

[35] Grujičić M., Jovičić-Bata J., Rađen S., Novakovic B., Šipetić-Grujičić S. (2016). Work motivation and job satisfaction of health workers in urban and rural areas. Vojnosanit. Pregl..

[36] Gilles I., Burnand B., Peytremann-Bridevaux I. (2014). Factors associated with healthcare professionals’ intent to stay in hospital: A comparison across five occupational categories. Int. J. Qual. Health Care.

[37] Australian Institute of Health and Welfare (2022). Rural and Remote Health. https://www.aihw.gov.au/reports/rural-remote-australians/rural-and-remote-health.

[38] Australian Institute of Health and Welfare (2022). Health Workforce. https://www.aihw.gov.au/reports/workforce/health-workforce.

[39] CCmedical (2018). What’s the Difference between Rural and Remote Doctor Work?. https://www.ccjobs.com.au/blog/whats-the-difference-between-rural-and-remote-doctor-work.

[40] Satuf C., Monteiro S., Pereira H., Esgalhado G., Marina Afonso R., Loureiro M. (2018). The protective effect of job satisfaction in health, happiness, well-being and self-esteem. Int. J. Occup. Saf. Ergon..

[41] Marinucci F., Majigo M., Wattleworth M., Paterniti A.D., Hossain M.B., Redfield R. (2013). Factors affecting job satisfaction and retention of medical laboratory professionals in seven countries of Sub-Saharan Africa. Hum. Resour. Health.

[42] Mathauer I., Imhoff I. (2006). Health worker motivation in Africa: The role of non-financial incentives and human resource management tools. Hum. Resour. Health.

[43] Dieleman M., Cuong P.V., Anh L.V., Martineau T. (2003). Identifying factors for job motivation of rural health workers in North Viet Nam. Hum. Resour. Health.

[44] Friedberg M.W., Chen P.G., Van Busum K.R., Aunon F., Pham C., Caloyeras J., Mattke S., Pitchforth E., Quigley D.D., Brook R.H. (2014). Factors affecting physician professional satisfaction and their implications for patient care, health systems, and health policy. Rand Health Q..

[45] Barac A., Krnjaic P., Vujnovic N., Matas N., Runjic E., Rogoznica M., Markic J., Jelicic Kadic A. (2021). The impact of the COVID-19 pandemic on resident physicians: A cross-sectional study. Work.

[46] Coto J., Restrepo A., Cejas I., Prentiss S. (2020). The impact of COVID-19 on allied health professions. PLoS ONE.

[47] Cucinotta D., Vanelli M. (2020). WHO Declares COVID-19 a Pandemic. Acta Bio-Med. Atenei Parm..

[48] Jazieh A.R., Coutinho A.K., Bensalem A.A., Alsharm A.A., Errihani H., Mula-Hussain L., Al-Sukhun S., Sampaio-Filho C.A., Khorshid O., De Guzman R.B. (2021). Impact of the COVID-19 Pandemic on Oncologists: Results of an International Study. JCO Glob. Oncol..

[49] Bernacki K., Keister A., Sapiro N., Joo J., Mattle L. (2021). Impact of COVID-19 on patient and healthcare professional attitudes, beliefs, and behaviors toward the healthcare system and on the dynamics of the healthcare pathway. BMC Health Serv. Res..

[50] Luo M., Guo L., Yu M., Jiang W., Wang H. (2020). The psychological and mental impact of coronavirus disease 2019 (COVID-19) on medical staff and general public—A systematic review and meta-analysis. Psychiatry Res..

[51] Xiong Y., Peng L. (2020). Focusing on health-care providers’ experiences in the COVID-19 crisis. Lancet Glob. Health.

[52] Shi L., Song K., Rane S., Sun X., Li H., Meng Q. (2014). Factors associated with job satisfaction by Chinese primary care providers. Prim. Health Care Res. Dev..

[53] Savageau J.A., Ferguson W.J., Bohlke J.L., Cragin L.J., O’Connell E. (2011). Recruitment and retention of primary care physicians at community health centers: A survey of Massachusetts physicians. J. Health Care Poor Underserv..

[54] Schoen C., Osborn R., Doty M.M., Squires D., Peugh J., Applebaum S. (2009). A Survey of Primary Care Physicians in Eleven Countries, 2009: Perspectives on Care, Costs, and Experiences: Doctors say problems exist across all eleven countries, although some nations are doing a better job than others. Health Aff..

[55] Veall M.R., Zimmermann K.F. (1996). Pseudo-R2 measures for some common limited dependent variable models. J. Econ. Surv..

[56] Hosmer D.W., Lemeshow S., Sturdivant R.X. (2013). Applied Logistic Regression.

[57] Jones A.M. (2012). Models for Health Care.

[58] Murray L., Nguyen H., Lee Y., Remmenga M., Smith D.W. Variance inflation factors in regression models with dummy variables. Proceedings of the Conference on Applied Statistics in Agriculture.

[59] Chen Y., You Y., Wang Y., Wang Y., Dai T. (2022). Global Insights into Rural Health Workers’ Job Satisfaction: A Scientometric Perspective. Front. Public Health.

[60] Rostami F., Babaei-Pouya A., Teimori-Boghsani G., Jahangirimehr A., Mehri Z., Feiz-Arefi M. (2021). Mental Workload and Job Satisfaction in Healthcare Workers: The Moderating Role of Job Control. Front. Public Health.

[61] Yi M., Jiang D., Wang J., Zhang Z., Jia Y., Zhao B., Guo L., Chen O. (2022). Relationships among thriving at work, organisational commitment and job satisfaction among Chinese front-line primary public health workers during COVID-19 pandemic: A structural equation model analysis. BMJ Open.

[62] Stobbe E.J., Groenewegen P.P., Schäfer W. (2021). Job satisfaction of general practitioners: A cross-sectional survey in 34 countries. Hum. Resour. Health.

[63] Kagan I., Hendel T., Savitsky B. (2021). Personal initiative and work environment as predictors of job satisfaction among nurses: Cross-sectional study. BMC Nurs..

[64] Barili E., Bertoli P., Grembi V., Rattini V. (2022). Job Satisfaction among Healthcare Workers in the Aftermath of the COVID-19 Pandemic (No. 22/04). HEDG, c/o Department of Economics, University of York. https://www.york.ac.uk/media/economics/documents/hedg/workingpapers/2022/2204.pdf.

[65] Pincus J.D. (1986). Communication Satisfaction, Job Satisfaction, And Job Performance. Hum. Commun. Res..

[66] Vermeir P., Degroote S., Vandijck D., Mariman A., Deveugele M., Peleman R., Verhaeghe R., Cambré B., Vogelaers D. (2017). Job Satisfaction in Relation to Communication in Health Care among Nurses: A Narrative Review and Practical Recommendations. SAGE Open.

[67] Loughman T.P., Snipes R.L., Pitts J.P. (2009). The effects of physicians’ communication satisfaction and their perceptions of empowerment on their likelihood to recommend a hospital to their peers. Manag. Res. News.

[68] Zerden L.d.S., Lombardi B.M., Richman E.L., Forte A.B., McCollum M. (2021). Addressing Burnout among the Frontline Healthcare Workforce during COVID-19: A Scoping Review & Expert Interviews. J. Health Hum. Serv. Adm..

[69] Wilson M.M., Devasahayam A.J., Pollock N.J., Dubrowski A., Renouf T. (2021). Rural family physician perspectives on communication with urban specialists: A qualitative study. BMJ Open.

[70] Hein S. (1996). EQ for Everybody: A Practical Guide to Emotional Intelligence.

[71] Gardiner M., Sexton R., Kearns H., Marshall K. (2006). Impact of support initiatives on retaining rural general practitioners. Aust. J. Rural Health.

[72] De Kock J.H., Latham H.A., Leslie S.J., Grindle M., Munoz S.-A., Ellis L., Polson R., O’Malley C.M. (2021). A rapid review of the impact of COVID-19 on the mental health of healthcare workers: Implications for supporting psychological well-being. BMC Public Health.

[73] Iyengar K.P., Jain V.K., Vaishya R. (2022). Current situation with doctors and healthcare workers during COVID-19 pandemic in India. Postgrad. Med. J..

[74] Isautier J.M., Copp T., Ayre J., Cvejic E., Meyerowitz-Katz G., Batcup C., Bonner C., Dodd R., Nickel B., Pickles K. (2020). People’s Experiences and Satisfaction with Telehealth During the COVID-19 Pandemic in Australia: Cross-Sectional Survey Study. J. Med. Internet Res..

[75] Snoswell C.L., Caffery L.J., Haydon H.M., Thomas E.E., Smith A.C. (2020). Telehealth uptake in general practice as a result of the coronavirus (COVID-19) pandemic. Aust. Health Rev..

[76] Cashman H., Sushil S., Mayson E., Milliken S., Lavee O., Awford A., Hamad N. (2022). Telemedicine for rural and regional patient access to haematology services during the COVID-19 pandemic in Australia. Lancet Haematol..

[77] NSW Rural Doctors Network (2022). COVID-19 Support. https://www.nswrdn.com.au/site/covid-19.

[78] Czeisler M.É., Marynak K., Clarke K.E., Salah Z., Shakya I., Thierry J.M., Ali N., McMillan H., Wiley J.F., Weaver M.D. (2020). Delay or Avoidance of Medical Care Because of COVID-19–Related Concern—United States. MMWR Morb. Mortal. Wkly. Rep..

[79] Fung T.H.M., Kuet M.-L., Patel M.K., Puri P. (2021). Addressing COVID-19 fear to improve clinic attendance for patients with wet age-related macular degeneration. Acta Ophthalmol..

[80] Jessup R.L., Bramston C., Beauchamp A., Gust A., Cvetanovska N., Cao Y., Haywood C., Conilione P., Tacey M., Copnell B. (2021). Impact of COVID-19 on emergency department attendance in an Australia hospital: A parallel convergent mixed methods study. BMJ Open.

[81] Fisher K., Davey A.R., Magin P. (2022). Telehealth for Australian general practice: The present and the future. Aust. J. Gen. Pract..

[82] Haydon H.M., Snoswell C.L., Thomas E.E., Broadbent A., Caffery L.J., Brydon J.A., Smith A.C. (2021). Enhancing a community palliative care service with telehealth leads to efficiency gains and improves job satisfaction. J. Telemed. Telec..

[83] Salem R., El Zakhem A., Gharamti A., Tfayli A., Osman H. (2020). Palliative care via telemedicine: A qualitative study of caregiver and provider perceptions. J. Palliat. Med..

[84] Read Paul L., Salmon C., Sinnarajah A., Spice R. (2019). Web-based videoconferencing for rural palliative care consultation with elderly patients at home. Support. Care Cancer.

[85] Fraser S., Greenhalgh T. (2001). Complexity science: Coping with complexity: Educating for capability. BMJ.

[86] Martiniuk A., Colbran R., Ramsden R., Edwards M., Barrett E., O’Callaghan E., Bullock R., Lowe E.F., Karlson D., Curnow J. (2020). Capability... What’s in a word? Rural Doctors Network of New South Wales Australia is shifting to focus on the capability of rural health professionals. Rural Remote Health.

[87] Sen A. (1979). Equality of What. The Tanner Lecture on Human Values. https://www.suz.uzh.ch/dam/jcr:ffffffff-df42-7cac-ffff-ffffd4ec9ff2/SEN_1.pdf.

[88] López Barreda R., Robertson-Preidler J., Bedregal García P. (2019). Health assessment and the capability approach. Glob. Bioeth..

[89] Leßmann O., Bonvin J.-M. (2011). Job-satisfaction in the broader framework of the capability approach. Manag. Rev..

[90] Ramsden R., Pit S., Colbran R., Payne K., Tan A.J.H., Edwards M. (2022). Development of a framework to promote rural health workforce capability through digital solutions: A qualitative study of user perspectives. Digit. Health.

[91] Stephenson J., Yorke M. (1998). Capability and Quality in Higher Education.

[92] Nelson T., Johnson S., Bebbington P. (2008). Satisfaction and burnout among staff of crisis resolution, assertive outreach and community mental health teams. Soc. Psychiatry Psychiatr. Epidemiol..

