# Supplementary material for: Job Satisfaction of Health Practitioners Providing Outreach Health Services during COVID-19 in Rural New South Wales (NSW) and the Australian Capital Territory (ACT), Australia
Source: Healthcare (Basel). 2022 Dec 20;11(1):3. doi: 10.3390/healthcare11010003 (PMC9819187; doi:10.3390/healthcare11010003)
Supplement: Supplementary file 1 [file healthcare-11-00003-s001.zip › healthcare-1965175-supplementary.pdf]

USING EXISTING DATA COLLECTIONS IN RESEARCH:

GUIDELINES FOR RESEARCHERS

INTRODUCTION

Sometimes, researchers wish to conduct a research project that uses an existing set of data which was previously collected for some other purpose.

The data may come from a previous research project, or may be routine data from an educational institution, healthcare provider, government department or authority, or private sector organisation. This existing dataset may have been collected by someone else or by the researcher themselves in another role.

For instance, a clinician may wish to use their patients' medical records, a teacher may wish to use students' assessment results, or a creative practitioner may wish to use video footage that was obtained as part of producing a creative work.

Such projects raise particular ethical issues around privacy and consent, and the use of these datasets may be governed by state or federal privacy legislation. These guidelines will highlight some specific issues around consent, requirements for ethical approval and privacy legislation that you need to be aware of if you would like to conduct a project with an existing dataset.

We suggest that you work your way through the flow chart below, which will guide you through relevant considerations to an “outcome” which will uncover some of the things you should consider when planning your project.

The information following the flowchart is designed to help you work your way through the questions by providing definitions and other relevant information. This information is guidance only and does not constitute legal advice. If you are unsure you should seek specific advice regarding your particular circumstances.

FOR FURTHER INFORMATION, CONTACT:

Ethics Office
P: +61 2 8627 8111
E: ro.humanethics@sydney.edu.au

Using existing data collections in research: use this flowchart to find out what you should consider when planning your project.

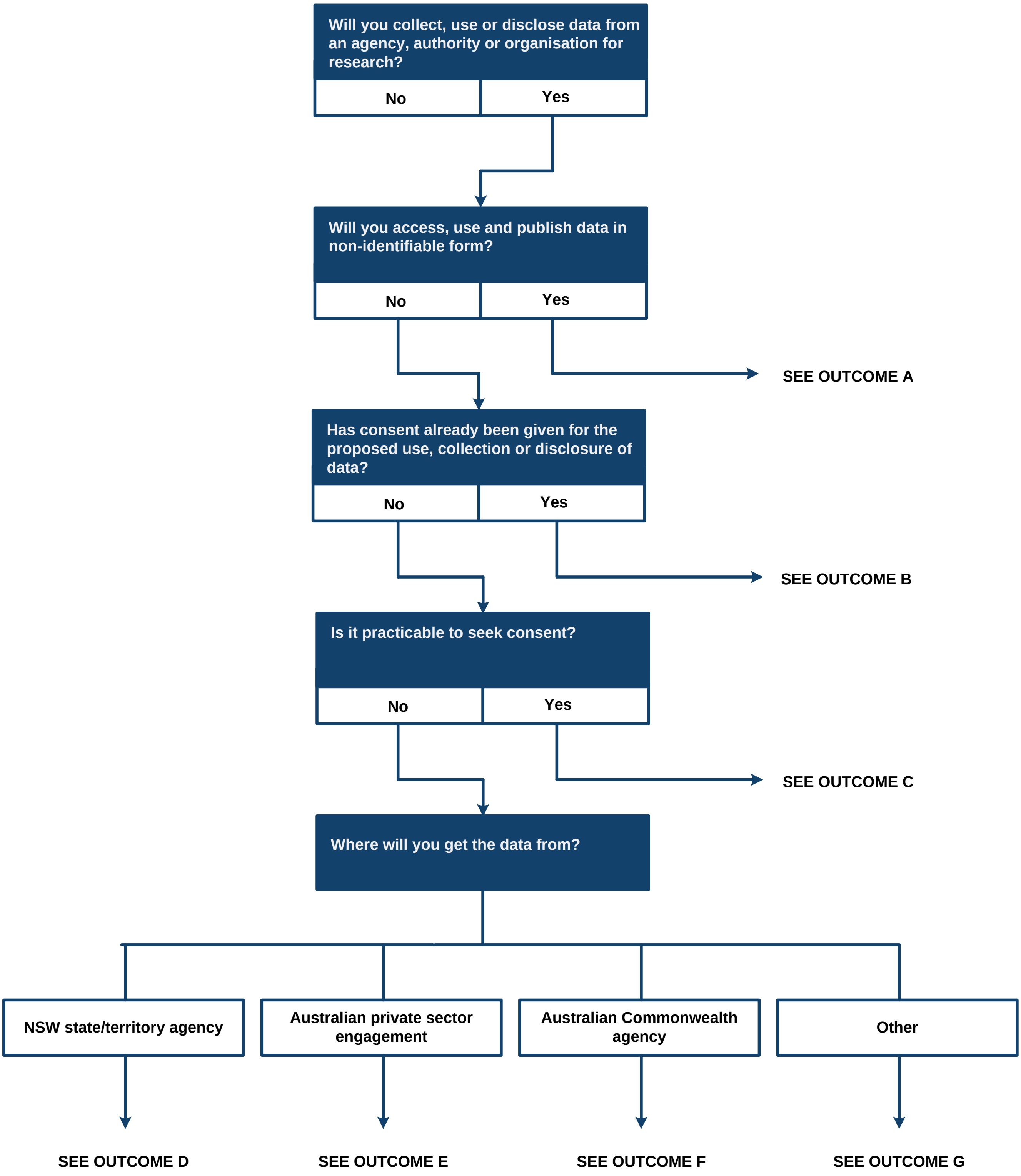

OUTCOME A

HREC APPROVAL: You should check with the Ethics Office and the place (e.g. journal) where you wish to publish your findings whether ethical approval is required for your project. Depending on the circumstances, ethical approval may not be required to do research with existing non-identifiable datasets.

CONSENT: If you will access the data in **non-identifiable** form, there is no requirement to seek consent from the people concerned as this would be impossible.

PRIVACY LEGISLATION: non-identifiable data is not considered “personal information” and therefore privacy legislation is not relevant to your project.

OUTCOME B

HREC APPROVAL: You will need HREC approval for your project.

CONSENT: You do not need to seek consent again, provided that consent has already been given for the use, collection or disclosure that you are proposing (by all relevant individuals) and it is reasonable to expect this consent to cover the current project. The HREC will assess whether it is reasonable for the existing consent to cover your new project when they review your application.

PRIVACY LEGISLATION: You should make sure you abide by relevant privacy legislation when collecting, using or disclosing this information in your research project. . Depending on the source of the original data, and the proposed use and disclosure, this may include NSW State privacy legislation or the Federal *Privacy Act 1988* (or both).

OUTCOME C

HREC APPROVAL: You will need HREC approval for your project.

CONSENT As it is considered practicable to seek consent from the people concerned, you should do so. This will need to take place after you have received HREC approval. You could consider employing an opt-out approach to consent in order to minimise burdens on participants and potential selection biases. If you adopt this approach, in your ethics application you will need to explain how your project satisfies the criteria in section 2.3.6 of the *National Statement.*

PRIVACY LEGISLATION: You should ensure you abide by relevant privacy legislation when making contact with these individuals to solicit their consent. Depending on the source of the original data, and the proposed use and disclosure, this may include NSW State privacy legislation or the Federal *Privacy Act 1988* (or both).

OUTCOME D

HREC APPROVAL: You will need HREC approval for your project.

CONSENT: As it is not considered practicable to seek consent from the people concerned, you will need to apply to the HREC for a waiver of consent. In your ethics application, you need to explain how your project satisfies the criteria for a waiver of consent in section 2.3.10 of the *National Statement on Ethical Conduct in Human Research*.

LEGAL RESTRICTIONS: The collection, use or disclosure of personal information from a NSW state agency, without consent from the people concerned, is governed by state privacy legislation. Where this is for research purposes, it can only be permitted under the following exemptions/directives:
- **Health information** –*Statutory Guidelines on Research* under the *Health Records and Information Privacy Act*.
- **Non health information** – This direction from the Privacy Commissioner under the Privacy and Personal Information Protection Act.

OUTCOME E

HREC approval: You will need HREC approval for your project.

CONSENT: As it is not considered practicable to seek consent from the people concerned, you will need to apply to the HREC for a waiver of consent. In your ethics application, you need to explain how your project satisfies the criteria for a waiver of consent in section 2.3.10 of the *National Statement on Ethical Conduct in Human Research*.

LEGAL RESTRICTIONS: The collection, use or disclosure of personal information from an Australian private sector organisation, without consent from the people concerned, is governed by the federal *Privacy Act 1988.* Where this is for research purposes, it can only be permitted under the following exemptions/directives:
- **Health information** –*Guidelines Approved Under Section 95A of the Privacy Act.*
- **Non-health information** – the collection, use or disclosure of non-health information without consent may not be permissible by law, unless a specific exemption under the *Privacy Act 1988* applies.

OUTCOME F

HREC APPROVAL: You will need HREC approval for your project.

CONSENT: As it is not considered practicable to seek consent from the people concerned, you will need to apply to the HREC for a waiver of consent. In your ethics application, you need to explain how your project satisfies the criteria for a waiver of consent in section 2.3.10 of the *National Statement on Ethical Conduct in Human Research*.

LEGAL RESTRICTIONS: The collection, use or disclosure of personal information for research purposes from an Australian Commonwealth agency, without consent from the people concerned, is governed by the federal *Privacy Act 1988.* Where this is for research purposes, it can only be permitted under the following exemptions/directives:
- **Medical research** –*Guidelines Approved Under Section 95 of the Privacy Act*.
- **Non-medical research** – the collection, use or disclosure of personal information for non-medical research may not be permissible by law, unless a specific exemption under the *Privacy Act 1988* applies.

OUTCOME G

HREC APPROVAL: You will need HREC approval for your project.

CONSENT: As it is not considered practicable to seek consent from the people concerned, you will need to apply to the HREC for a waiver of consent. In your ethics application, you need to indicate how your project satisfies the criteria for a waiver of consent in section 2.3.10 of the *National Statement on Ethical Conduct in Human Research*.

LEGAL RESTRICTIONS: The collection, use or disclosure of personal information obtained from other jurisdictions for research purposes, without consent from the people concerned, may be governed by relevant local legislation. You should confirm with relevant authorities whether there are any legal restrictions prior to submitting your ethics application. Australian privacy legislation may also apply if the information sourced from other jurisdictions relates to individuals protected by Australian privacy legislation and is used or disclosed within Australia.

UNDERSTANDING THE OUTCOMES

For some of the flowchart outcomes, which legislation is relevant to your project will depend on the type of information concerned or the type of research you are doing. If you aren't sure which category your project falls into, these definitions might help.

- **Is it health information?** For the purposes of some state and federal privacy legislation, it is important to work out whether the information contained in the datasets you will be using in your research is health information or not. As defined here, **health information** is personal information which pertains to a person's health or disability, their use or desired use of health services, the donation of their body parts, or genetic information in a form that is or could be predictive of the health of the individual or their genetic relatives. It includes any personal information collected by a health service provider during the course of providing treatment and care to an individual.

- **Am I doing medical research?** For the purposes of some federal privacy legislation, it is important to work out whether the research you are doing is medical research or not. **Medical research** includes epidemiological studies, interventions aiming to improve health, clinical/medical record audits that will be published, etc. Please note that your project may be a medical research project even if the personal information you are using/collecting/disclosing is not health information (e.g. if you are collecting demographic information for the purposes of a medical research project).

WORKING YOUR WAY THROUGH THE FLOWCHART

Will I use, collect or disclose data from an agency, authority or organisation for research?

For the purposes of these guidelines, we are interested in the collection, use or disclosure of information from agencies, authorities and organisations for research purposes.

Let's first examine what we mean by use, collection or disclosure of data for research. If you want to know more, you can find a full definition of these terms here.

- **Collection** refers to gathering, acquiring or obtaining information from any source and by any means, either directly from the individual concerned or indirectly from a third party.
- **Disclosure** refers to the release of personal information to other third parties outside the agency/organisation that collected it. It does not include giving individuals information about themselves.
- **Use** refers to the handling and management of information. In research, this includes data cleaning, data analysis, inclusion of information in a publication etc.

It's important to understand that any time an agency, authority or organisation discloses information to a third party, that third party becomes a collector of the information. Therefore, a disclosure of information by organisation A is also a collection of information by third party B.

This includes government departments, bodies and authorities as well as private sector organisations. For the purposes of relevant Australian privacy legislation, it does not include individuals acting in their personal capacity.

It is important to remember that this may apply to your project even if you collected the data yourself, because you may be acting as part of an agency, authority or organisation. For example, data collected by an academic at the University of Sydney is considered to be data collected by a NSW state agency, and data collected by a clinician at a private hospital is considered to be data collected by a private sector organisation.

Will I access/use the data in non-identifiable form?

Where existing datasets are used in research, the identifiability of the data has important implications. The *National Statement on Ethical Conduct in Human Research* categorises data into three forms:

- **Identifiable data** is data from which individual people can be identified, because it contains 'identifiers' (e.g. names, dates of birth, images, addresses).
- **Re-identifiable data** is data in which identifiers have been removed and replaced by a code. So individuals can only be identified by people with access to the code.
- **Non-identifiable data** is data from which individuals cannot be identified, because identifiers were never present or have been removed.

In privacy legislation, identifiable and re-identifiable information is referred to as 'personal information' and is subject to special legal protections.

In Australia, strict state and federal legislation governs its use, collection and disclosure although in some cases there are special provisions concerning research. Personal information is also subject to special protections in research ethics, such that researchers have additional responsibilities in terms of consent and ethical approval for personal information. So it is preferable that you access and use your dataset in non-identifiable form if possible.

However, it may not be possible for you to access the data in non-identifiable form (e.g. if you are the person who collected and holds the data and it is in an identifiable form), or you may need to access and use it in identifiable or re-identifiable form to achieve your research aims (e.g. if you are conducting data linkage or need

identifiers to satisfy your research aims).

As outlined here, you should bear in mind that identifiers are contextually dependent. That is, the possibility of identification depends on a number of factors such as the other information in the dataset and the other information available to the person who has the data.

This means you need to think carefully when determining whether your dataset is non-identifiable. For example, a dataset is re-identifiable to someone who has access to the code, but non-identifiable to someone who does not. Further, some information which does not fall into the traditional category of 'identifiers' may identify individuals in some contexts. For instance, one or more rare characteristics (e.g. a disease diagnosis, company and employment title) may serve to identify an individual, especially if the person accessing the dataset has relevant background knowledge.

You should also bear in mind that what matters here is the form in which you will *use, access or disclose* the data, not just the form you will publish it in. Outcome A only applies if each of the access, use and publication of the data is in non-identifiable form. You should answer 'no' if one of the criteria are not met (for example, the data is identifiable when you access it but you intend to publish it in a non-identifiable form).

Was consent given for the proposed use, collection or disclosure?

You may have already envisaged at the time of collecting your data that you would want to use or disclose it for research purposes later on, and sought consent from all of the people concerned for this future use in research.

For example, you might have collected the data as part of a previous research project and sought consent at that time for use in future unspecified research projects such as this one. Alternatively, you may be interested in using data from a healthcare service that asks patients at the time of admission whether they are willing for their clinical information to be used in research. If this is the case, you should answer "Yes" to this question.

You should be careful to consider, however, whether the consent that was given reasonably covers the particular use or disclosure you are proposing in this project, whether it is still reasonable to operate under that consent if a long period of time has elapsed or their capacity to consent is likely to have changed, and whether all of the people whose data you wish to use gave their consent. These factors will be considered by the HREC when assessing your proposal. If not, you may wish to consider continuing down the "No" pathway.

Is it practicable to seek consent?

Informed and voluntary consent form the foundation of ethical human research by protecting and respecting human autonomy. Therefore, you should attempt to seek informed and voluntary consent from the people whose information is contained in the dataset if possible.

However, there are some situations where it would not be practical or possible to seek consent, or where seeking consent may place people at risk or compromise the validity of your results. For example, there may be so many people in the dataset that it would be impractical to contact them all, there may be a risk of distress associated with contacting certain people about your research (e.g. if it relates to a sensitive topic or you would be contacting people whose relatives had died), their contact details may be missing or out of date, or there may be a risk of selection bias associated with seeking consent and thus reduced validity of your research findings. In that case, it may be more appropriate to answer "No" here and continue down the "waiver of consent" path, whereby you carry out the research without individual people's consent after getting permission from a HREC.

Where am I getting the data from?

It's important that you identify where you will be getting your data from, because the legal implications of collecting, using, or disclosing data for research purposes are impacted by where it is being sourced from. In some cases, it may not be legally permissible to collect, use or disclose data for research purposes. For convenience, we've divided the possible sources into four categories:

- **NSW state agency/authority:** a NSW public sector agency, including a government department or the Teaching Service, statutory body representing the Crown, the NSW Police Force, Service NSW Division of the Government Service, local government authority etc. The University of Sydney is a NSW state agency/ authority. Find a full definition here.
- **Australian private sector organisation:** an Australian individual, body corporate, partnership, other unincorporated association or trust that is not a small business operator, registered political party, state or Commonwealth agency or authority. Find a full definition here.
- **Australian Commonwealth agency/authority:** a Minister, Department, body or tribunal established under a Commonwealth act for a public purpose, including a body established by the Governor General, a person holding office under a Commonwealth act, a federal court, the Federal Police, a Norfolk Island agency, etc. Find a full definition here.
- **Other:** other sources of information include international agencies, authorities and organisations and Australian state/Territory agencies outside NSW.

It is important to note that privacy legislation applies to each stage of the data lifecycle (collection, use and disclosure). Therefore, more than one set of legislative requirements may apply if the information is collected from one jurisdiction/source but used and disclosed in another.
